# Discontinuing Hemodialysis with Patient Care and a Successful 9-Year Follow-up in a Patient Presumed to have End-Stage Kidney Disease Scheduled to Lifelong Hemodialysis: A Case Report

**DOI:** 10.3390/clinpract11010019

**Published:** 2021-02-26

**Authors:** Omer Toprak, Emel Aslan Bozyel, Burak Alp

**Affiliations:** 1 Department of Medicine, Division of Nephrology, Balikesir University School of Medicine, 10145 Balikesir, Turkey; 2 Department of Medicine, Division of Internal Medicine, Balikesir University School of Medicine, 10145 Balikesir, Turkey; emelaslan006@hotmail.com (E.A.B.); burakalp316@gmail.com (B.A.)

**Keywords:** patient care, hemodialysis, end-stage kidney disease, renal function recovery

## Abstract

End-stage kidney disease patients who require hemodialysis for more than 3 months have a small chance of leaving dialysis unless they have a kidney transplant. Educating the patient about lifestyle changes can play a major role in improving kidney function. Therefore, we created a patient education program according to our nephrology experiences. Herein, we show an end-stage kidney disease patient who underwent hemodialysis for 6 months. Afterwards, dialysis was terminated with patient care, and the patient was then followed up for 9 years without dialysis. To date, there have been no reports regarding the termination of long-term dialysis with a kidney care program and the ensuing 9-year follow-up without renal replacement therapy.

## 1. Introduction

About 2.5 million patients worldwide require dialysis or kidney transplants. Patients who need renal replacement therapy are also increasing day-by-day [1]. It is unfortunately impossible to accommodate such a large number of renal replacement therapy patients. As such, millions of patients die because they are not able to have dialysis or find a donor candidate for kidney transplantation. At this point, it is very important to educate patients and their caregivers about chronic kidney disease (CKD), which will hopefully postpone the need for dialysis or kidney transplantation. Kidney care programs play a role in kidney function [2,3,4,5], including Toprak’s Kidney Care, which provides patient and caregiver trainings, suggests lifestyle changes, promotes regular nutrition and exercise, supplies adequate drugs in proper doses, improves morale and motivation, regulates fluid status, preserves residual kidney function, and offers nephroprotective alternative medicines.

In this paper, we present a patient evaluated to have end-stage kidney disease (ESKD), as well as diabetic nephropathy and an arteriovenous fistula. Thus, permanent vascular access was included in a lifelong dialysis program. However, the patient was removed on patient care after 6 months of hemodialysis and did not need hemodialysis for 9 years afterward. Although this case report is a single case, it is promising because it emphasizes that long-term dialysis treatment can be terminated with careful and strict patient care. Further, these patients can live healthily for many years. The start of dialysis should not be a barrier to research the possibility of discontinuing dialysis in CKD patients who have enough urination.

## 2. Case Report

In March 2010, a 71-year-old patient applied to the nephrology outpatient clinic because of hemodialysis regulation. We hospitalized the patient for nephrological evaluation. In our medical history, we found that the patient had suffered from hypertension for 10 years, type 2 diabetes mellitus for 15 years, and ESKD due to diabetic nephropathy for 2 years. In addition, the patient and her caregiver were noted to have clinical depression and anxiety. Before starting dialysis, the patient had a hemodialysis certified specialist for 20 months, and an arteriovenous fistula was placed in her left arm. Hemodialysis started in September 2009 due to uncontrolled hypertension and a glomerular filtration rate (GFR) of 8.4 mL/min/1.73 m^2^.

She underwent hemodialysis for 6 months, 3 times a week, and 4 h in each session. The day the patient was admitted to us was the patient’s routine dialysis day. She was stable and had 800 cc/day of urine. A laboratory analysis revealed anemia, hypopotassemia, hypomagnesemia, hypophosphatemia, hypoalbuminemia, mild acidosis, and an eGFR of 11.2 mL/min/1.73 m^2^ (Table 1). On the renal ultrasound, we found a bilateral normal-sized parenchymal thickness and bilateral grade 1 echogenicity kidneys. We started to correct the patient’s electrolyte abnormalities and malnutrition by changing the meditation and suggesting new lifestyle and nutritional habits according to Toprak’s Kidney Care (Table 2 and Table 3). We added sodium bicarbonate, erythropoietin, multi-vitamins, magnesium, vitamin D, and calcium acetate to the treatment and adjusted the insulin dose. During hemodialysis, the dry weight of the patient was determined to be 65 kg. Approximately 1 L of fluid was ultrafiltrated in each session, and the patient was allowed to consume a total of 700 cc of water and 300 cc of other liquids. However, because there was no hypervolemia, we allowed her to drink 1.5 L of water and 500 cc of other fluids per day. We did not take the patient on dialysis for 6 days in the hospital because of clinical and laboratory findings. As expected, an increase in serum urea and creatinine values was detected after 6 days. Moderate hyponatremia was also detected on the sixth day of her admission. Therefore, we reduced her daily water intake to 1250 cc. We did not find any uremic symptoms, encephalopathy, acidosis, uncontrolled hypertension, hyponatremia, hyperphosphatemia, hyperpotassemia, or fluid overload, and the urine output was sufficient. GFR levels were fixed at about 9 mL/min/1.73 m^2^ (Table 1). There was no indication of hemodialysis. The depression and anxiety of the patient and her caregiver improved. According to the clinical and laboratory findings, we decided to end dialysis on 8 March 2010. We followed-up our patient for 9 years without dialysis.

During this follow-up period, GFR values increased up to 30.8 mL/min/1.73 m^2^. The patient and her caregiver attended our training seminars. Seven years later, we found that the bilateral small (85 mm vs. 75 mm) kidneys showed decreased parenchymal thickness (9 mm vs. 10 mm). During the follow-up, the patient was hospitalized 4 times. Three of these hospitalizations were caused by a resistant urinary tract infection, while one was due to pneumonia and hyponatremia. During these hospitalizations and acute infectious periods, acute kidney injury (AKI) occurred only during the patient’s first hospitalization (29 July 2015). The creatinine value increased from 1.6 mg/dL to 2.1 mg/dL and then regressed to 1.9 mg/dL after appropriate medical treatment. There was no reduction in the urine output. There were no major adverse cardiac events. All hospitalizations and treatments were managed by the same nephrologist. In each outpatient clinic appointment, we reviewed whether our recommendations for eating, drinking, lifestyle, and medication use were implemented by the patient through a standard questionnaire. Our patient largely complied with the exact serving sizes we suggested during the 9-year follow-up period. The patient lived for 9 years without the need for dialysis. The caregiver who brought the patient to the hospital died in 2019, and we lost contact with the patient after the last control in March 2019, when the patient was 81 years old.

## 3. Discussion

Despite improved dialysis technology and treatment, mortality rates for hemodialysis patients are still high. The survival of elderly patients on maintenance dialysis has been found to be only 26.6 months [1].

Most patients diagnosed with AKI can leave hemodialysis within the first 6 months [6]. However, before the patient applied to us, she was diagnosed with dialysis-dependent ESKD, which was secondary to the diabetic nephropathy diagnosed by a medical doctor in a state hospital who specialized in hemodialysis and internal medicine. The diagnosis of diabetic nephropathy was based on clinical findings. We could not find a renal biopsy. Moreover, the patient received pre-dialysis care for 20 months before starting dialysis. In addition, a permanent vascular access, an AV fistula, was created during this pre-dialysis care in preparation for hemodialysis. Under normal conditions, the first dialysis initiation is not wrong for a patient with an ESKD diagnosis due to diabetic nephropathy, who has uncontrolled hypertension, and whose GFR value is below 10 mL/min/1.73 m^2^. However, based on the patient’s medical documents and data, we could not conclude whether the decision of the chronic hemodialysis program was incorrect or too early for the present case. Many patients with CKD may develop AKI and are erroneously labeled as having reached ESKD and becoming dialysis-dependent [5,7,8]. Perhaps the present case had AKI on CKD and had dialysis at an early stage. For the patient, the eGFR at the start of dialysis was 8.4 mL/min/1.73 m^2^, which may be considered as ESKD.

The reasons that caused us to terminate our patient’s hemodialysis treatment are multifactorial. In our case, one of the most important factors when terminating dialysis was the regulation of the patient’s fluid balance. The continued hemodialysis was accompanied by a reduction in residual kidney function and a progressive deterioration in kidney function. Many patients that may have a potential for renal function recovery (RFR) are missed because of incorrect measurements of ideal dry weight, unnecessary fluid restriction, excessive use of diuretics, or lack of monitoring of residual kidney function [1,5,7,8]. Most of these reasons may be valid for our patient. 

In our patient, daily fluid intake was restricted during the 6-month period when she underwent hemodialysis, and fluid was removed from her body via ultrafiltration in each dialysis session. All of these are factors could lead to a further increase in renal perfusion disorder, which may be present in elderly patients such as ours. Moreover, it could decrease the amount of urine and impair renal functions. After increasing the patient’s oral fluid intake, removing ultrafiltration, and ending hemodialysis, a significant increase was observed in our patient’s urine amount (Table 1). 

In addition, appropriate nutrition, lifestyle changes, rational drug use, and alternative treatments may be among the beneficial factors effective in terminating dialysis in our patient. We increased the duration of exercise, corrected the disturbed sleep pattern, enabled her to sunbathe and siesta, and treated the depression and anxiety without using medication. The patient stopped her high salt intake, and kidney-friendly spices, bitter, and lemon were added instead. Consumption of frozen or canned meats, processed meats, mineral water, and salty pickles were halted and replaced with kidney-friendly drinks, soups, and meats. We banned white baked goods and white bread, and normalized low vitamin D and magnesium levels. 

**Figure 1:**
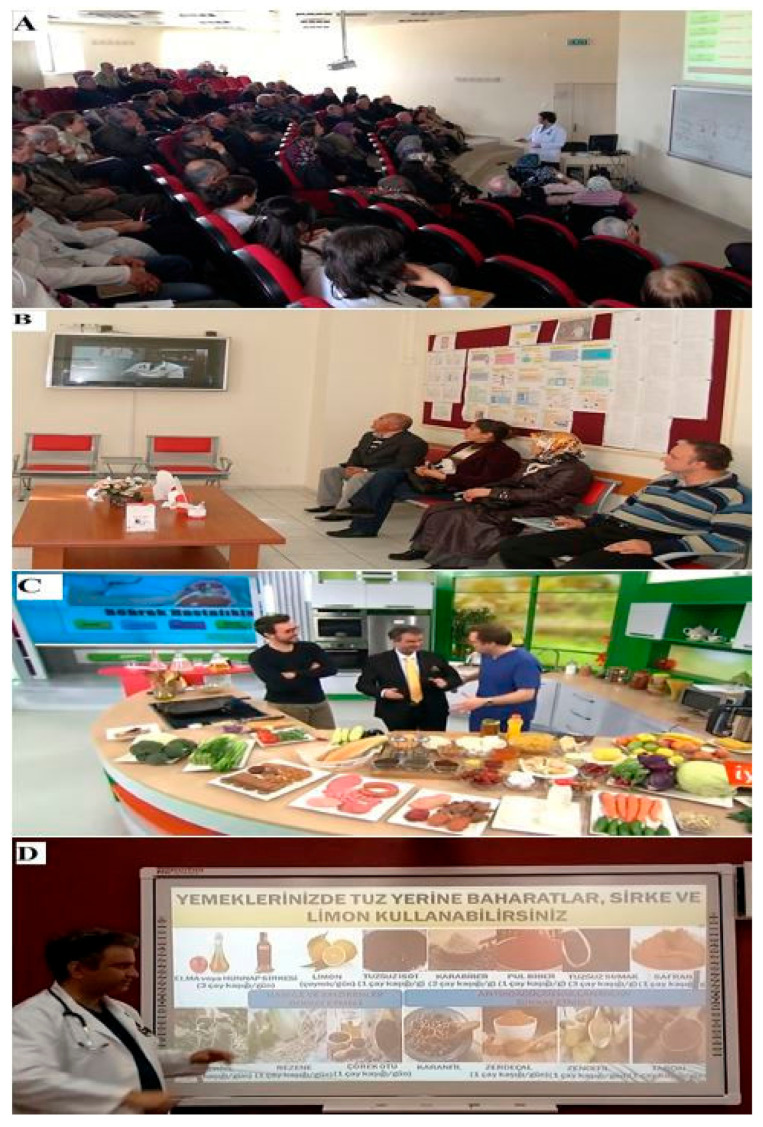
(**A**) One of the educational patient and caregiver conferences at the hospital; (**B**) nephrology outpatient clinic education television; (**C**) one of the national television programs about patient and caregiver education and diet; and (**D**) one of the educational social media videos for patients and caregivers. All of these videos and conferences above were made by and belong to us. The person seen in the video thumbnail is the corresponding author of the case report. We obtained permission from the patients and the television program’s production company to use the screen capture. The source of the TV program is TRT-1 TV.

All follow-up examinations and tests of the patient were performed by the same nephrologist. In addition, we provided spiritual support to our patient and her caregiver, training them through our care program (Figure 1). In our case, the potential pathomechanisms behind the recovery of renal function may have been caused by better control of diabetes and hypertension. Further, recovery may have resulted from restricting a high salt died, increasing exercise, and raising the patient’s fluid intake. All of these may have a positive effect on renal function, as they decrease oxidative stress and proteinuria, and increase renal perfusion. In addition, some alternative treatments that we provided to the patient—such as special sauerkraut and black seed oil—may have had a positive effect on increasing renal perfusion, decreasing oxidative stress, proteinuria, creatinine, and urea levels, and may have had a positive effect on renal recovery [9,10]. Previous studies have indicated that probiotic consumption may improve gastrointestinal function and slow CKD progression [9]. Moreover, black seed oil may protect kidneys in patients with diabetic nephropathy such as our patient [10]. 

Another interesting aspect of the case is that before the patient applied to us, she stated to her dialysis doctor that she wanted to come to us in order to reduce or stop the dialysis. The medical doctors in the dialysis center said that it would be suicidal to make such an attempt; dialysis, the doctor argued, could only end when one received a kidney transplant. He advised her to stay away from medical charlatans. However, contrary to what our colleagues say, we followed-up the patient for 9 years without dialysis. If this patient we are presenting had not applied to us, she would have probably continued dialysis.

What are the barriers preventing the discontinuation of hemodialysis in clinical practice? We indicated several, as follows: the lack of RFR knowledge; false belief that ESKD patients could never discontinue dialysis; not evaluating the possibility of RFR of the patient by nephrologists, particularly when a patient transfers to a new outpatient center; a lack of the extra effort needed to obtain medical records pertaining to dialysis initiation; financial reasons; the dialysis industry; a lack of optimal care needed for AKI patients who continue to require dialysis after hospital discharge; no guidelines regarding RFR monitoring in dialysis patients [5,7,8,9,10,11]. There are studies that support the above considerations. Numerous case reports and registries have cited RFR delays in dialysis-dependent patients. According to recent studies and large-scale registries, 0.3% to 8% of patients with ESKD receiving long-term dialysis recover some degree of kidney function, allowing for the discontinuation of dialysis for a varying period of time and even permanently [1,7,8,11,12,13,14].

This case is just the tip of the iceberg. Thousands of patients are on dialysis unnecessarily. It is an extremely dangerous and risky situation to decide to stop dialysis treatment in a presumed ESKD patient because of the risk of death. Naturally, nobody wants to take this risk. There are no guidelines for the cessation of dialysis. In our case, before terminating our patient’s dialysis, serum creatinine values were high and around 5 mg/dL on average. If the patient we removed from dialysis had had any complications such as a brain hemorrhage or a heart attack, we could perhaps have been accused of malpractice. Unfortunately, about 2 weeks after we terminated the patient’s dialysis, the doctors at the dialysis center complained to the nephrology association and requested them to open an investigation against us, saying that we risked the patient’s life by removing her from dialysis. 

This is where the importance of Toprak’s Kidney Care comes into play. Secondary to our 17 years of nephrology experience, we have created a care program for CKD patients known as Toprak’s Kidney Care [5]. The corresponding author of this study has been working as the only nephrologist for 14 years in the hospital due to the lack of academic staff. For this reason, the follow-up and treatment of all patients are carried out by the same nephrologist. This leads to a stronger connection with the patients and their caregivers, increased control of the nephrologist over their patients and patient follow-up files, and more successful patient follow-ups. In addition, the corresponding author of the study is also experienced and trained in nutrition and spiritual care for CKD patients. The diets of the patients, their calorie intake, daily protein, carbohydrate, and fat needs, as well as their fluid and electrolyte balance, are regulated by the nephrologist who cares for the patients. With the spiritual support given in this case, the depression and anxiety of the patient and her caregiver were eliminated without the need for medication. This also may have played a role in the success of patient follow-up. The fact that approximately 7000 pre-dialysis patients have been followed-up by us (the GFR value of most of these patients is below 15 mL/min) has given us a great deal of experience in pre-dialysis patient follow-up and has led to the emergence of Toprak’s Kidney Care. We could not find any other study or information in the literature examining so many pre-dialysis patients who were followed-up by a single nephrologist. As a result of our experience, we use many new practices that are not currently available in the literature, such as a special probiotic sauerkraut, black seed oil, and jujube tea (Table 3). As a result of their use, we have observed that patients experience improved renal function. We hope that we can continue to make clearer recommendations regarding these alternative practices as a result of placebo-controlled studies.

We organize a patient’s lifestyle, medications, and diet so that they do not enter dialysis or enter dialysis late, and we train patients and their relatives with Toprak’s Kidney Care. Most patients starting dialysis do not receive adequate kidney care beforehand [13]. Although the present case received pre-dialysis care for 20 months, we saw that she had many issues such as high salt intake, consumption of processed foot, and frozen or canned meats, etc. One of the most important features of Toprak’s Kidney Care is that we not only educate the patients but we also check whether our recommendations are implemented. In each outpatient clinic appointment, we review in detail whether our guidelines for eating, drinking, lifestyle, and medication are implemented using a standardized questionnaire. We closely follow the patients not only in terms of nephrology but also comorbid diseases. When necessary, we hospitalize our patients and request consultation from relevant branches. For example, in this case, infections requiring hospitalization were successfully treated in a nephrology clinic. We attach importance not only to the care of the patient but also to the education of the caregiver, as in our case. The more patients and caregivers know about CKD, the more they avoid habits that can damage the kidneys. This situation may cause patients to have better kidney function. As such, we are in contact with our patients 24/7. In addition, we hold question and answer sessions with patients throughout the day. Our patients do not use any medication or supplements without our approval. Nephrotoxic drug use, excessive salt consumption, and smoking are almost nonexistent in patients in our care program. 

The conferences for CKD patients and their caregivers that we organize every month at the hospital are also useful for educating patients. We explain almost everything they need to eat and drink, as well as what they need to do for a healthy life. On March 5th 2020, we held our 133rd education conference. We share these conferences on social media with live broadcast for patients who cannot attend. We also use local and national TV channels to educate patients. We prepared 18 educational videos for CKD patients and their caregivers based on thousands of questions asked by patients about lifestyle, eating and drinking habits, and medication use. By sharing these videos on social media, we enable our patients to watch them at home. In the training videos, we use a language that patients can easily understand and not jargon-heavy medical language. In addition, while the patients and their caregivers wait for examination at the hospital, they also watch our training videos on our training television in the waiting room (Figure 1). No fees are charged to patients or their relatives for all these educational activities. We do our job fondly and reflect this love to our patients and their caregivers. In summary, we use every opportunity we have for the education of our patients.

Many patients with CKD caused by diabetic nephropathy do not have small kidneys, as the present patient did [15]. In our case, a reduction in kidney size was seen on a control ultrasound. When we removed our patient from dialysis, there was no improvement in kidney function, and GFR values were still low. There are no reports in the literature reporting that dialysis was terminated using a patient care program. Only one study showed that three CKD patients with a low protein diet had RFR and cessation of dialysis [12].

The limitation of this case study is that the treatment approaches we used here may not have the same effect on every patient. The strength of this case is that we demonstrated that a dialysis patient can be removed from dialysis and integrated into a patient care program, thus enabling the discontinuation of dialysis despite decreased kidney function.

## 4. Conclusions

The possibility of terminating hemodialysis in patients with ESKD who have a sufficient urine output should not be ignored. Further, patient care may also be considered during the termination of dialysis in these patients. Toprak’s Kidney Care may be considered during the recovery of kidney function and dialysis discontinuation in patients with residual kidney function to avoid unnecessary, complicated, and expensive dialysis.

## Figures and Tables

**Table 1:** Renal, metabolic, nutritional, and fluid load parameters of the patient.

	First Start to HD	HD Stopped	HD Free 3 Days	HD Free 6 Days	HD Free 1 Year	HD Free 3 Years	HD Free 7 Years	HD Free 9 Years
Date	2 September 2009	3 March 2010	5 March 2010	8 March 2010	1 April 2011	25 February 2013	30 January 2017	26 March 2019
Renal Parameters								
Creatinine, mg/dL	4.9	3.8	4.8	4.7	2.5	2.1	2.0	2.2
Urea, mg/dL	158	52	94	144	118	113	109	115
eGFR, mL/min/1.73 m^2^	8.4	11.2	8.5	8.7	18.6	22.6	23.3	20.5
Urine amount, mL/day	1500	800	1200	1600	1700	1500	2000	1700
Blood pressure, mmHg	210/120	130/70	130/70	130/70	100/60	130/80	150/80	150/70
Urine P/C, mg/mg		180		220	140	510	1137	4097
Blood pH	7.26	7.31	7.36	7.33	7.42	7.38	7.44	
Blood HCO_3,_ mEq/L	16	19	21	20	23	22	22	
Parathormone, pg/mL		192			283	214	157	169
Sodium, mEq/L	128	142	139	129	140	149	141	139
Potassium, mEq/L	4.9	3.0	3.8	3.7	5.8	5.4	4.8	4.5
Calcium, mg/dL	7.8	8.4	7.6	7.1	7.4	8.4	9.4	8.7
Phosphorus, mg/dL	5.9	1.4	2.8	3.0	5.2	3.9	3.7	3.9
Magnesium, mg/dL		1.6		1.8	3.2	2.0	2.1	2.0
Kidney Ultrasound								
Linear dimension, mm		100/92					85/75	
Echogenity (grade)		1/1					1/1	
Parenchymal thickness		15/15					9/10	
Volume Parameters								
Pretibial oedema	+/+	–/–	–/–	–/–	–/–	–/–	–/+	++/++
Pleural effusion, cm	0/0	0/0		0/0	0/0			0/0
Pericardial effusion, cm		0		0	0			0
Cardiothoracic ratio	0.51	0.47		0.50	0.48		0.50	
Metabolic Parameters								
BMI, kg/m^2^	27.05	25.15	25.34	25.15	24.77	27.05	27.05	28.58
Weight, kg	71	66.0	66.5	66.0	65.0	71.0	71.0	75.0
Hemoglobin, mg/dL	9.3	10.4	9.9	9.5	12.8	11.2	13.2	10.8
Albumine, gr/dL	2.7	2.8		3.2	3.7	4.2		3.9
Uric acid, mg/dL	8.9	8.0		6.7	7.6	6.2	7.3	7.0
Glucose, mg/dL	157	142	67	113	118	129	167	109
HbA_1_C, %		6.1			7.1	6.4	8.0	
HDL-C, mg/dL		41				74		
LDL-C, mg/dL		72				133		
Triglyceride, mg/dL		107				111		

Abbreviations: HD—hemodialysis; eGFR—estimated glomerular filtration rate, estimated via the CKD-EPI (chronic kidney disease epidemiology collaboration) equation; urine P/C—urine protein to creatinine ratio; NA—not available; LVEF—left ventricular ejection fraction; BMI—body mass index; HDL-C—high-density lipoprotein cholesterol; LDL-C—low-density lipoprotein cholesterol.

**Table 2:** Educational, clinical, and lifestyle parameters of the patient secondary to patient care.

	Basal	Day 3	Day 6	Year 1	Year 3	Year 5	Year 7	Year 9
	Hemodialysis-Free Time (9 Years)							
Educational/Patient Related Activities								
Patient and caregiver education, total hours	0	6	12	24	48	72	96	110
Spiritual support, total number	0	1	2	5	11	17	23	29
Approval from nephrologist in all drugs	–	+	+	+	+	+	+	+
All controls with the same nephrologist	–	+	+	+	+	+	+	+
Hospitalization for any reason, number	1	0	0	0	0	2	2	0
Major adverse cardiac events, number	0	0	0	0	0	0	0	0
Medications Used by Patient								
Sodium carbonate, 500 mg 3 times/day	–	+	+	+	+	+	+	+
Multivitamin (B, C, folat), twice/week	–	+	+	+	+	+	+	+
Darbepoetin alfa, 20 to 50 mcg/week	–	+	–	+	+	–	–	+
Calcitriol 0.25 mcg/day if necessary	–	+	+	+	+	+	+	+
Calcium acetate, 500 mg 3 times/day	–	–	+	+	+	–	–	+
Magnesium oxide, 365 mg once/day	–	+	+	+	+	–	–	–
Allopurinol, 150 mg twice/week	–	–	–	+	–	+	–	–
Paracetamol, 500 mg once/week	–	–	–	+	+	+	+	+
Insulin glargine, 12 to 6 units/once-daily	+	+	+	+	+	+	+	+
Furosemide 20 to 40 mg twice/week	–	–	–	–	–	+	+	+
Lacidipine, 4 mg/day	–	–	–	–	–	+	+	+
Polystyrene sulfonate, 880 mg/day	–	–	–	+	–	–	–	–
Nephrotoxic antibiotics or herbals	–	–	–	–	–	–	–	–
Non-steroidal anti-inflammatory drugs	–	–	–	–	–	–	–	–
Iodinated contrast use without prophylaxis	–	–	–	–	–	–	–	–
Depression/Anxiety								
Beck depression score (patient)	34			7	6		5	
Beck anxiety score (patient)	42			6	6		4	
Beck depression score (caregiver)	30			5	5		5	
Beck anxiety score (caregiver)	32			4	4		5	
Lifestyle Changes								
Exercise, minutes/day	5	10	15	30	40	45	45	45
Sleeping, hours/day	4	4	5	7	7	8	8	8
Sunbathe, 30 min, 3 times/week	–	–	+	+	+	+	+	+
Smoking or alcohol intake	–	–	–	–	–	–	–	–
High salt intake, >4 g/day	+	–	–	–	–	–	–	–

Abbreviations: BAI—Beck anxiety inventory (8–15: mild, 16–25: moderate, 26–63: severe); BDI—Beck depression inventory (10–18: mild, 19–29: moderate, 30–63: severe).

**Table 3:** Eating and drinking habits of the patient secondary to patient care.

	Basal	Day 3	Day 6	Year 1	Year 3	Year 5	Year 7	Year 9
	Hemodialysis-Free Time (9 Years)							
Probiotic Consumption								
Homemade yogurt, 35 g/day	–	–	–	+	+	+	+	+
Apple or jujube vinegar, 3 mL/day	–	–	–	+	+	+	+	+
Special sauerkraut ^a^, 30 g/day	–	–	–	+	+	+	+	+
Fruit Consumption								
Green sour apple, 90 g/day	–	+	+	+	+	+	+	+
Watermelon, 200 g/week	+	+	+	+	+	+	+	+
Lemon, 120 g/week	+	+	+	+	+	+	+	+
Fruits other than those listed above	+	–	–	–	–	–	–	–
Meat Consumption								
Beef, lamb, or mutton, 300 g/week	+	+	+	+	+	+	+	+
Processed, frozen, or canned meats	+	–	–	–	–	–	–	–
Natural and organic chicken, 100 g/week	–	–	–	+	+	+	+	+
Salmon, anchovy, or mackerel, 100 g/month	+	–	–	–	–	+	+	+
Meat or chicken bouillon	+	–	–	–	–	–	–	–
Vegetable Consumption								
Raw greens (lettuce, arugula, parsley, etc.)	+	–	–	–	–	–	–	–
Boiled and drained vegetables	–	+	+	+	+	+	+	+
Roasted and dehydrated pepper, eggplant, tomato, onion, and garlic, 2 pieces/week	–	–	+	+	+	+	+	+
Cultivated/red pine mushrooms, 100 g/week	–	–	+	+	+	+	+	+
Spice and Bitter Consumption ^c^								
Unsalted green olives, 20 g/day	–	–	–	+	+	+	+	+
Unsalted isot, black or chili pepper, saffron, fennel, ginger, turmeric, or cinnamon, 2 g/day	–	–	–	+	+	+	+	+
Unsalted sumac or rosemary, 6 g/day	–	–	–	+	+	+	+	+
Cold pressed black seed oil, 1 g/day	–	–	–	+	+	+	+	+
Ketchup, mayonnaise, or salted pickles	+	–	–	–	–	–	–	–
Minth and thyme	+	–	–	–	–	–	–	–
Drinking Habits								
Daily alkaline water (pH 7.5–8.5 mL)	1000	1500	1500	1750	1750	1750	1500	1750
Fizzy drinks, 3 in 1 coffee, mineral water	+	–	–	–	–	–	–	–
Linden tea or black tea, 150 mL/day	+	+	+	+	+	+	+	+
Green tea, 200 mL/week	–	–	–	+	+	+	+	+
Fruit or vegetable juices other than apple	+	–	–	–	–	–	–	–
Boiled/drained vegetable soup ^b^, 100 mL/day	–	+	+	+	+	+	+	+
Lentil, chickpea, ezogelin, or tarhana soups	+	–	–	–	–	–	–	–
Meat and bone broth soups, 100 mL/day	–	–	–	+	+	+	+	+
Other Nutritional Habits								
Egg white, 3 times/day	–	+	+	+	+	+	+	+
Egg yolk, twice/week	–	+	+	+	+	+	+	+
Unsalted/unroasted pumpkin seeds, 10 g/day	–	–	+	+	+	+	+	+
Hazelnut, walnut, almond, or cashew 150 g/week	–	–	+	+	+	+	+	+
Unsalted siyez bread, 56 g/day	–	–	–	+	+	+	+	+
White bakery products or white bread	+	+	+	–	–	–	–	–
Corn, sunflower, or hazelnut oil/margarine	+	+	+	–	–	–	–	–
Cold pressed olive oil, 28 g/day	+	–	–	+	+	+	+	+
Processed sugar, dry legumes, or pollen	+	–	–	–	–	–	–	–
Unsalted curd/cottage/tongue cheese, 30 g/day	–	–	–	+	+	+	+	+

^a^ Boiled and drained white cabbage, apple cider vinegar, olive oil, lemon, garlic, and water are used for preparation. ^b^ One serving per day any of the following: spinach, leek, zucchini, artichoke, broccoli, purslane, white cabbage, celery, or okra. ^c^ Maximum three types of spice and bitters can be used on the same day.

## Data Availability

All data are included in the manuscript.
